# Updated risk models for lung cancer due to radon exposure in the German Wismut cohort of uranium miners, 1946–2018

**DOI:** 10.1007/s00411-023-01043-2

**Published:** 2023-09-11

**Authors:** M. Kreuzer, V. Deffner, M. Sommer, N. Fenske

**Affiliations:** Federal Office for Radiation Protection, Ingolstaedter Landstr. 1, 85764 Neuherberg, Germany

**Keywords:** Radon, Lung cancer, Epidemiology, Cohort, Risk

## Abstract

UNSCEAR recently recommended that future research on the lung cancer risk at low radon exposures or exposure rates should focus on more contemporary uranium miners. For this purpose, risk models in the German Wismut cohort of uranium miners were updated extending the follow-up period by 5 years to 1946–2018. The full cohort (*n* = 58,972) and specifically the 1960 + sub-cohort of miners first hired in 1960 or later (*n* = 26,764) were analyzed. The 1960 + sub-cohort is characterized by low protracted radon exposure of high quality of measurements. Internal Poisson regression was used to estimate the excess relative risk (ERR) for lung cancer per cumulative radon exposure in Working Level Months (WLM). Applying the BEIR VI exposure-age-concentration model, the ERR/100 WLM was 2.50 (95% confidence interval (CI) 0.81; 4.18) and 6.92 (95% CI < 0; 16.59) among miners with attained age < 55 years, time since exposure 5–14 years, and annual exposure rates < 0.5 WL in the full (*n* = 4329 lung cancer deaths) and in the 1960 + sub-cohort (*n* = 663 lung cancer deaths), respectively. Both ERR/WLM decreased with older attained ages, increasing time since exposure, and higher exposure rates. Findings of the 1960 + sub-cohort are in line with those from large pooled studies, and ERR/WLM are about two times higher than in the full Wismut cohort. Notably, 20–30 years after closure of the Wismut mines in 1990, the estimated fraction of lung cancer deaths attributable to occupational radon exposure is still 26% in the full Wismut cohort and 19% in the 1960 + sub-cohort, respectively. This demonstrates the need for radiation protection against radon.

**Supplementary Information:**

The online version contains supplementary material available at 10.1007/s00411-023-01043-2.

## Introduction

The United Nations Scientific Committee on the Effects of Atomic Radiation (UNSCEAR) has recently reviewed the radon-related lung cancer risk in epidemiological studies (UNSCEAR 2020). They concluded that in miners studies the relationship between cumulative exposure to radon and relative risk of lung cancer is approximately linear and that the linear increase is additionally modified by time since exposure, attained age and exposure rate. The preferred risk model is thus a model including these three modifiers. The lifetime excess absolute risk (LEAR) of lung cancer per WLM was calculated for several cohort studies of miners based on the BEIR VI exposure-age-concentration model, using a mixed male/female population and exposure scenario of 2 WLM from age 18 to 64 years (UNSCEAR 2020). The resulting LEARs ranged from 2.4 (Wismut cohort) to 7.5 (Eldorado cohort) × 10^*−*4^ per WLM, and represent an important database for the epidemiological approach for radon dose conversion. The variability of LEARs across the studies offers different possibilities of dose conversion, which led to some controversial discussions in the International Commission on Radiological Protection (ICRP) (Harrison et al. 2020, 2021; Laurier et al. 2020; Marsh et al. 2021). The LEARs from epidemiological studies depend—among other factors—to a large extent on the risk model derived from the different studies of miners. In order to improve risk models, UNSCEAR (2020) recommended that future research on the lung cancer risk at low radon exposure or exposure rates should focus on time periods with the best available exposure assessment to reduce measurement error and should consider age- and time-related effect modifiers, exposure rate and, if possible, potential confounders.

For this purpose, the 1960 + sub-cohort of German uranium miners (Wismut miners) was updated; this sub-cohort includes only miners hired in 1960 or later with protracted exposure to low radon concentrations, which has been assessed based on radon measurements. In previous risk analyses this sub-cohort (Kreuzer et al. 2015, 2018) was characterized by a relatively young age, which hampered a valid estimation of effect modifiers at older ages or longer times since exposure. Due to the extended mortality follow-up by 5 years to the end of 2018, the proportion of deceased individuals in this sub-cohort increased from 19.3% to 25.1%, and the number of lung cancer deaths from 495 to 663. The larger number of deaths, longer time since exposure and older attained age together with the availability of data on important confounders (e.g. smoking, occupational exposure to silica dust and external gamma radiation) allow to further improve risk models at low exposures and exposure rates. Two types of risk models were estimated for the 1960 + sub-cohort and for comparison with previous results also for the full cohort: (1) parametric models including time since median exposure and age at median exposure as continuous variables and exposure rate as categorical variable (Tomasek et al. 2008; Kreuzer et al. 2018), (2) the categorical BEIR VI exposure-age-concentration model as used in the pooled study of the 11 miners cohorts (NRC 1999), UNSCEAR (2009, 2020) and the new PUMA study (Pooled Uranium Miners Analysis) (Richardson et al. 2022; Kelly-Reif et al. 2023). For both types of models, the relative risk was predicted for the exposure scenario of 2 WLM at age 18 to 64 over attained age up to 94 years as in other publications (Tirmarche 2010; UNSCEAR 2020) and the corresponding LEARs were calculated. In addition, differences in risk estimates between the full cohort and the 1960 + sub-cohort were discussed.

## Methods

### Study population

The German cohort of uranium miners has been described previously (Kreuzer et al. 2010, 2018). The full cohort includes 58,972 men employed for at least 180 days in the Wismut company in former Eastern Germany in the operation period from 1946 to 1990, the 1960 + sub-cohort includes 26,764 men hired for the first time in 1960 or later. Mortality follow-up has been extended by 5 years to the end of 2018 (i.e., 31/12/2018). Vital status was provided by local registration offices. Causes of death were obtained from death certificates and autopsy files from the Wismut pathology archive. In addition to new follow-up data, 359 previously missing causes of death from 1955 to 2013 were successfully traced through extensive searches in archival documents, many of them from the 1960s. In the present analyses, the cohort entry date was defined as the date of first employment plus 180 days (inclusion criterion), and the exit date was defined as the earliest date of death, loss to follow-up, or end of follow-up.

The early period of mining (1946–1955) at the Wismut company was characterized by high radon exposures due to lack of radiation protection measures and lack of radon measurements. In 1955, ambient air measurements of radon gas started in the different mines and from 1955 to 1958 the radon concentrations sharply decreased due to introduction of ventilation measures in the mines (Appendix Fig. 1). Annual cumulative exposure to radon progeny in Working Level Months (WLM: concentration of short-lived radon progeny per litre of air that gives rise to 1.3 × 10^5^ MeV of alpha-particle energy after complete decay for 1 month (170 h) = 3.5 mJ h m^−3^) was retrospectively assessed for each miner via a comprehensive job-exposure matrix (JEM). For each mining facility, workplace (underground, open pit, milling or surface) and calendar year the exposure to radon progeny in WLM was determined by an expert group for scientific purposes (HVBG 2005). The JEM was based on ambient measurements, if available, or, for years without measurements (particularly for the early mining period from 1946 to 1954), on expert ratings considering the first available ambient measurements of radon gas in later years, uranium deposit and delivery, ventilation and mine architecture over time.

Information on smoking habits were extracted from the Wismut health archives, mainly based on data from the regular medical check-ups which had been introduced in 1970. In these documents, the current smoking habits were given in predefined categories for each year. This only allows the definition of three rough smoking categories for risk analyses in the 1960 + sub-cohort: “non-smoker” (in all years “non-smoker”), “moderate/heavy smoker” (if in any year the classification “more than 5 years of smoking or more than 10 cigarettes smoked per day” was indicated) and “light smoker” (for all other specifications such as “occasional smoker”, “less than 5 years or less than 10 cigarettes smoked per day”, “cigar/pipe smoked”). In order to be comparable to previous risk analyses (Kreuzer et al. 2015, 2018) and due to the small number of lung cancer deaths among non-smokers, the categories non-smoker and light smoker were combined. Data on smoking were available for 56% of the 1960 + sub-cohort.

### Statistical modelling

Typical statistical methodology was applied to model radon-related lung cancer risks by internal Poisson regression (NRC 1999; Kreuzer et al. 2018; Richardson et al. 2022; Tomasek et al. 2008; Walsh et al. 2010), and two different model types were fitted: parametric models with continuous age- and time-related effect-modifying variables and the BEIR VI exposure-age-concentration model. For this purpose, individual data was first converted into grouped datasets to tabulate person-years at risk and lung cancer deaths in categories. Two grouped datasets were created, one for each model type. The following basic cross-classifications were used in both datasets: age *a* in 16 categories (0–14, 15–19, 20–24, …, 85 + years), calendar year in 15 categories (1946–1949, 1950–1954, …, 2005–2009, 2010–2014, 2015–2018), duration of employment *d* in three categories (0− < 5, 5– < 15, ≥ 15 years) calculated in a time-varying way, and start of employment in two categories (1946–1959, 1960–1989) to allow separate modelling of the 1960 + sub-cohort. Further categorization was model-specific and is described below.

For both model types, lung cancer mortality rates were assumed to follow an excess relative risk (ERR) model with the general structure:

$$r(a,y,d,w,...) = r_{0} (a,y,d) \times [1 + ERR(w,...{ )]}$$

Here, the mortality rate *r(a,y,d,w,…)* depends on attained age *a*, calendar year *y*, cumulative 5 year lagged exposure to radon progeny *w*, and potential further variables (indicated by “…”). It is expressed as the internal baseline mortality rate *r*_*0*_* (a,y,d)* stratified by age, calendar year and duration of employment, multiplied by an excess relative risk term *ERR(w,…)*. Note that technically an excess relative *rate* is modelled here, which is called excess relative *risk* in the following for simplification purposes. This term varied according to complexity and type of considered models. 95% Wald-type confidence intervals were calculated for the model parameters. All models were fitted for both, the full cohort and the 1960 + sub-cohort. Grouping of the datasets and statistical modelling was performed with the Epicure software.

#### Parametric models

In the first type of models, age- and time-related effect modification was modelled based on continuous variables. The grouped dataset was additionally cross-classified by cumulative 5 year lagged exposure to radon progeny *w* in nine categories (0, > 0– < 10, 10– < 50, 50– < 100, 100– < 200, 200– < 500, 500– < 1,000, 1,000– < 1,500, ≥ 1,500 WLM), exposure rate *er* in six categories (0– < 0.5, 0.5– < 1, 1– < 2, 2– < 4, 4– < 10, ≥ 10 WL), age at median exposure *e* in nine categories (0–19, 20–24, …, 55 + years), and time since median exposure *t* in 10 categories (0– < 5, 5– < 10, 10– < 15, …, ≥ 45 years) as in Kreuzer et al. (2018). Age at median exposure and time since median exposure referred to the point in time when one-half of the exposure cumulated up to a given date was reached and thus varied over time. Exposure rate was calculated as the total cumulative exposure (with a lag) divided by the total individual duration of exposure in months up to a given date, and thus represented a time-varying “average” exposure rate. In each cell of the grouped dataset, person-time weighted mean values of cumulative exposure, age at and time since median exposure were calculated and used as continuous variables in the parametric models.

Parametric models of different complexity were fitted:

Model 1 $$ERR\left( w \right) = \beta \,w$$

Model 2 $$ERR\left( w, e, t \right) = \beta \,w \times \exp \,[\alpha\,(\,e - 30) + \varepsilon \,(t - 20)]$$

Model 3 $$ERR(w,\,er,\,e,\,t) = \sum\nolimits_{j=1}^{6} {\beta_{j} } \,er_{j} \,w \times \exp \,[\alpha\,(e - 30) + \varepsilon (t - 20)]$$

In all models, *ß* quantifies the excess relative risk per unit of cumulative exposure to radon progeny *w* (and is in the following abbreviated with ERR/WLM or, in case of other scaling, with ERR/100 WLM). Model 1 denotes the “simple” linear ERR model. Model 2 contains exponential modifying effects for age at median exposure *e* (centered at 30 years) and time since median exposure *t* (centered at 20 years), with choice of centering values for comparability with previous results. Model 3 additionally contains exposure-rate specific estimates *ß*_*j*_ for cumulative exposure split based on six categories of exposure rate, these were defined by binary variables *er*_*j*_ for *j* = *1,…,6*. The models were selected and compared based on their deviances and likelihood ratio tests, as for example described in Richardson et al. (2022).

#### BEIR VI exposure-age-concentration model

The BEIR VI exposure-age-concentration model is based on categorical effect-modifying variables. For this model type, the grouped dataset contained cumulative 5 year lagged exposure to radon progeny split into four variables *w*_*5–14*_, *w*_*15–24*_, *w*_*25–34*_ and *w*_*35*+_, each with nine categories (0, > 0– < 10, 10– < 50, 50– < 100, 100– < 200, 200– < 500, 500– < 1000, 1000– < 1500, ≥ 1500 WLM), reflecting cumulative exposures received 5–14, 15–24, 25–34 and 35 and more years prior to a considered date, respectively. Exposure rate *er* was calculated in a similar way as described above, and classified in six categories (0– < 0.5, 0.5– < 1, 1– < 3, 3– < 5, 5– < 15, ≥ 15 WL) as in the pooled BEIR VI study (NRC 1999).

The following model was fitted:

Model 4 $$ERR\left( {w, \, a, \, er} \right) = \beta \,(\theta_{1} \,w_{5 - 14} + \theta_{2} \,w_{15 - 24} + \theta_{3} \,w_{25 - 34} + \theta_{4} \,w_{35 + } )\,\phi_{{{\text{age}}}} \,\gamma_{{{\text{rate}}}}$$

where *ß* represents the ERR/WLM in the reference category, since *θ*_*1*_ = 1 by definition. Parameters *θ*_*2,*_ *θ*_*3*_ and *θ*_*4*_ quantify effect modification by time since exposure. The parameters *ϕ*_age_ and *γ*_rate_ denote effect-modifying factors based on the representation of categorical variables with multiple binary variables and describe effects of categories of attained age (< 55, 55–64, 65–74 and 75 + years) and of exposure rates, respectively.

#### LEAR calculations

The lifetime excess absolute risk (LEAR) is the difference in lifetime risks for an individual from an exposed population $L{R}_{E}$ compared with an individual from an unexposed population $L{R}_{0}$ and is here approximated by

$$LEAR=L{R}_{E}-L{R}_{0}\approx \sum_{a={a}_{min}}^{{a}_{max}}{r}_{E}\left(a\right)S\left(a |{a}_{min}\right)-\sum_{a={a}_{min}}^{{a}_{max}}{r}_{0}\left(a\right)S\left(a |{a}_{min}\right)$$

where $S\left(a |{a}_{min}\right)=\mathrm{exp}(-\sum_{u={a}_{min}}^{a-1}{q}_{0}(u))$ is the probability to survive until age $a$ given survival to age ${a}_{min}$ with all-cause mortality rates ${q}_{0}\left(a\right)$ at age $a$. ${r}_{0}\left(a\right)$ is the baseline lung cancer mortality rate at age $a$ in absence of exposure. Likewise, ${r}_{E}(a)$ corresponds to the lung cancer mortality rate at age $a$ under exposure.

For the calculation of LEARs, attained age ${a}_{min}$ is set to $0$ to account for the full lifetime of an individual. The baseline lung cancer mortality rates ${r}_{0}(a)$ and all-cause mortality rates ${q}_{0}(a)$ are taken from the ICRP Euro-American-Asian mixed population (ICRP 2007) with ${a}_{max}=94$. The exposure scenario is 2 WLM from age 18 to 64 with a lag of $L=5$ years between age at exposure and age at actual risk amplification as used in Tomasek et al (2008). The terms for $ERR(\cdot )$ were chosen as described above. Note that the total LEAR can be obtained by multiplying the value for the LEAR per WLM with 94. All LEAR calculations were performed with the statistical software R (R Core Team 2022).

#### Interaction of radon and smoking

As in previous analyses (Kreuzer et al. 2018; UNSCEAR 2020; Leuraud et al. 2011), the following geometric mixture model (GMM) was fitted:

$$r\left( {a,y,d,w,s} \right) = r_{0} \,(a,y,d) \times [(1 + \beta \,w)\,\exp \,(\gamma \,s)]^{\lambda } \times [\beta \,w + \exp\,{ (\gamma \, s)]}^{1 - \lambda }$$

where *γ* describes the parameter associated with smoking category *s.* Depending on the choice of the mixing parameter λ, this model incorporates an additive (λ = 0) and a multiplicative model (*λ* = 1), as well as supra-additive/sub-multiplicative models (0 < *λ* < 1) and supra-multiplicative models (*λ* > 1). Here, models for a grid of values 0 ≤ *λ* ≤ 1.5 were compared based on the model deviances.

#### Sensitivity analyses

Sensitivity analyses in the models of the full cohort and the 1960 + sub-cohort considered (1) restriction to duration of employment for at least 5 years and (2) exclusion of open pit miners and millers. Potential confounding in the 1960 + sub-cohort was investigated by adjustment for cumulative exposure to external gamma radiation in mSv in an additive way and for smoking in a multiplicative way (Kreuzer et al. 2018). For sensitivity analyses, grouped datasets contained additional cross-classifications for workplace (four categories), 5 year lagged cumulative exposure to gamma radiation (eight categories) and smoking (three categories, as described above).

## Results

Table 1 provides a description of the cohorts. The mean duration of follow-up was 41.7 years and 39.6 years and corresponding person-years at risk 2,461,269 and 1,058,712 in the full cohort and 1960 + sub-cohort, respectively. While in the full cohort 57% of all cohort members were deceased by end of follow-up, this proportion was 25% in the 1960 + sub-cohort. The number of lung cancer deaths in the full cohort is appreciably higher than in the 1960 + sub-cohort (4329 versus 663). Notable is the more than fifteen times higher mean cumulative radon exposure in the full cohort compared to the 1960 + sub-cohort (280 WLM versus 17 WLM). This is mainly due to the extremely high average annual radon exposures in the years of operation before 1960 as illustrated in Appendix Fig. 1.

**Table 1:** Description of the full Wismut cohort and the 1960 + sub-cohort of miners first hired in 1960 or later, 1946–2018

Variable	Full cohort	1960 + sub-cohort
Persons, *n*	58,972	26,764
Person-years at risk	2,461,269	1,058,712
Mean duration of employment in years	13.4	10.1
Mean age at death in years	68	58
Mean age at end of follow-up	67	61
Mean duration of follow-up in years	41.7	39.6
Vital status, *n (%)*		
Alive at end of follow-up	23,330 (39.6)	19,457 (72.7)
Deceased	33,794 (57.3)	6719 (25.1)
Lost to follow-up	1848 (3.1)	588 (2.2)
Availability of cause of death, *n (%)*	32,411 (95.9)	6534 (97.2)
Lung cancer deaths, *n*	4329	663
Radon exposed miners, *n (%)*	50,759 (86.1)	22,571 (84.3)
Mean (Max) cumulative exposure in WLM	280 (3224)	17 (334)
Mean (Max) exposure rate^a^ in WL	2.95 (26.66)	0.23 (4.65)

*WLM* working level months

^a^Time-varying average exposure rate, calculated as the total cumulative exposure (without lag) divided by the total individual duration of exposure in months up to a given date

**Fig. 1:**
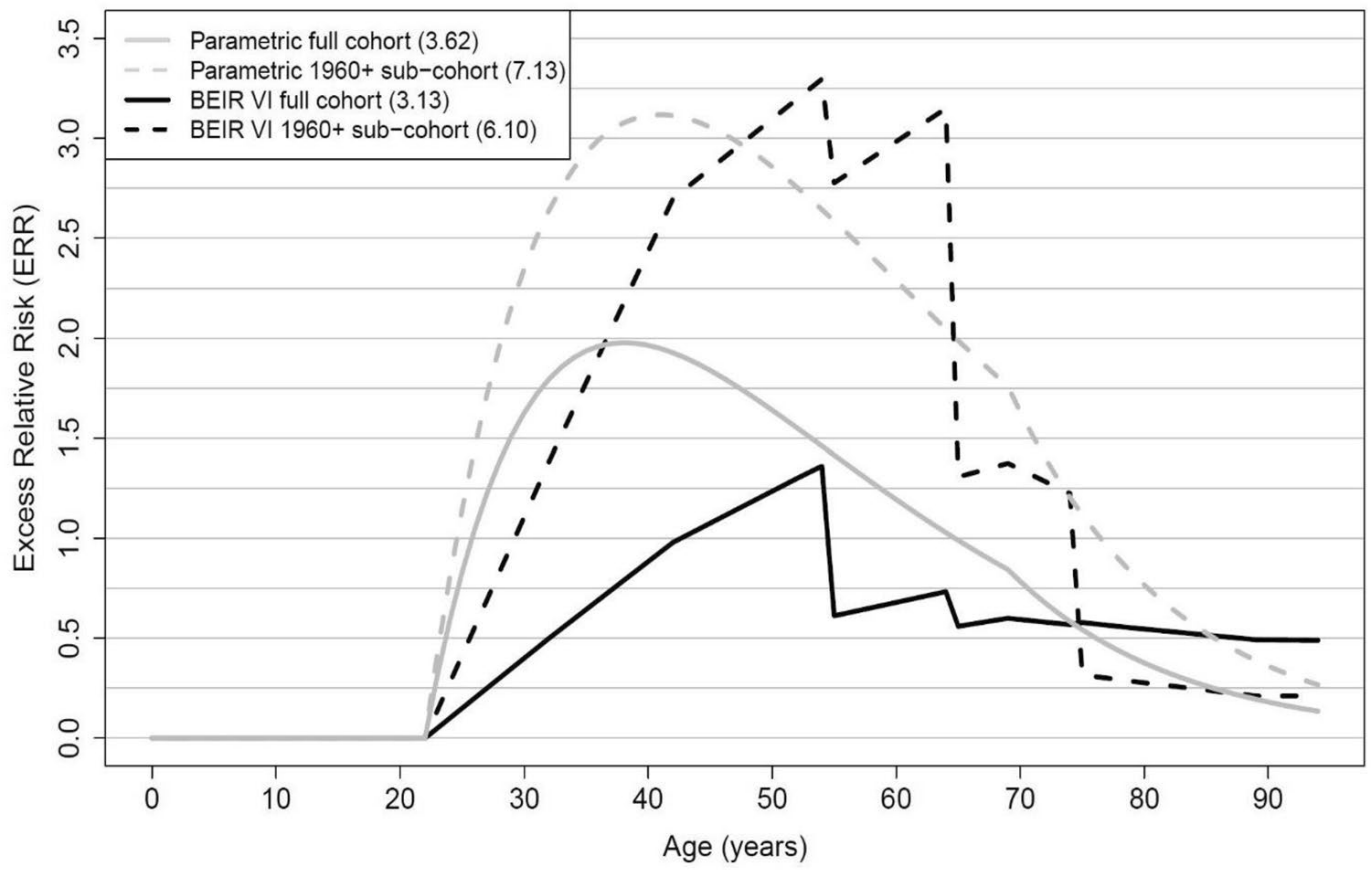
Excess relative risk predicted for different models in the full cohort and the 1960 + sub-cohort for the exposure scenario of 2 WLM from age 18 to 64 up to age 94 assuming a 5 year lag with corresponding total LEAR in brackets in figure legend (parametric full cohort: model 3, parametric 1960 + sub-cohort: model 2, BEIR VI full cohort and 1960 + sub-cohort: model 4, exposure-age-concentration model)

In Table 2, risk estimates based on parametric models (models 1–3) are given. Using a simple linear model, the ERR/100 WLM is 0.18 (95% CI 0.16; 0.21) in the full cohort and 1.34 (95% CI 0.75; 1.93) in the 1960 + sub-cohort, respectively. There is no overlap in both confidence intervals, indicating heterogeneity. The same holds true when model 2 was applied that takes additionally the two modifiers age at and time since median exposure into account. Model 2 provides a statistically significantly better fit than the simple linear model in both cohorts and is thus preferred. The ERR/WLM decreased with increasing age at median exposure and time since median exposure. Additional consideration of exposure rate in six categories in model 3 provides the best fit in the full cohort and is the finally preferred model for the full cohort, while no improvement of fit was found in the 1960 + sub-cohort, indicating that model 2 is the finally preferred model for the 1960 + sub-cohort. The inclusion of exposure rate has a strong influence on the lung cancer risk due to radon in the full cohort, showing a clear decrease in the ERR/100 WLM with increasing exposure rate, the so-called “inverse exposure-rate effect”. Although not statistically significant, this effect was also indicated in the 1960 + sub-cohort. The use of model 3 reduces heterogeneity between full cohort and 1960 + sub-cohort. The ERR/100 WLM at < 0.5 WL, centred at age at median exposure 30 years and time since median exposure 20 years is 2.83 (95% CI 1.57; 4.09) in the full cohort and 5.38 (95% CI 1.76; 8.99) in the 1960 + sub-cohort; both confidence intervals overlap, but the risk estimates differ by a factor of two. The LEARs per WLM for the finally preferred models are 3.62 × 10^–4^ (model 3) versus 7.13 × 10^–4^ (model 2) for the full and the 1960 + sub-cohort, respectively. The corresponding risk predictions for the exposure scenario of 2 WLM per year from age 18 to 64 years are given in Fig. 1.

**Table 2:** Radon-related lung cancer risk estimates according to parametric models applied to the Wismut full cohort and 1960 + sub-cohort

	Parameter	Full cohort	1960 + sub-cohort
Lung cancer deaths		4329	663
Person-years at risk		2,461,269	1,058,712
Model 1			
ERR/100 WLM (95% CI)	*ß*	0.18 (0.16; 0.21)	1.34 (0.75; 1.93)
LEAR per WLM (× 10^4^)		**0.82**	**6.09**
Model 2			
ERR/100 WLM^a^ (95% CI)	*ß*	0.53 (0.40; 0.66)	4.66 (1.71; 7.62)
Age at median exposure	exp(10*α*)	0.64 (0.54; 0.76)	0.74 (0.44; 1.26)
Time since median exposure	exp(10*ε*)	0.53 (0.46; 0.61)	0.47 (0.30; 0.73)
*p*-value (Model 2 vs. 1)		< 0.001	< 0.001
LEAR per WLM (× 10^4^)		**0.79**	**7.13**
Model 3			
ERR/100 WLM (95% CI)			
< 0.5 WL	*ß*_*1*_	2.83 (1.57; 4.09)	5.38 (1.76; 8.99)
0.5–1 WL	*ß*_*2*_	1.58 (0.91; 2.25)	4.66 (1.08; 8.23)
1–2 WL	*ß*_*3*_	1.13 (0.74; 1.52)	2.87 (< 0; 6.02)
2–4 WL	*ß*_*4*_	0.90 (0.63; 1.16)	–
4–10 WL	*ß*_*5*_	0.75 (0.55; 0.96)	–
10 + WL	*ß*_*6*_	0.48 (0.33; 0.63)	–
﻿ Age at median exposure	exp(10*α*)	0.60 (0.51; 0.72)	0.70 (0.41; 1.21)
﻿ Time since median exposure	exp(10*ε*)	0.48 (0.41; 0.55)	0.47 (0.30; 0.74)
﻿*p*-value (Model 3 vs. 2)		< 0.001	0.417
﻿LEAR per WLM (× 10^4^)		**3.62**	**7.83**

Values of LEAR per WLM (× 10^4^) (bold)

*ERR* excess relative risk, *CI* confidence interval, *WLM* working level months, *WL* working level

*p*-value of likelihood ratio test between two nested models

*LEAR *lifetime excess absolute risk (exposure of 2 WLM from age 18 to 64 years, maximum age 94 and ICRP Euro-American-Asian mixed population)

Baseline stratified by attained age, calendar year and duration of employment

^a^ERR/100 WLM for age at median exposure of 30 years and time since median exposure of 20 years

Table 3 shows the results of the risk analyses using the BEIR VI exposure-age-concentration model (model 4). The ERR/100 WLM at 5–14 years since exposure, < 55 years of attained age and < 0.5 WL exposure rate was 2.50 (95% CI 0.81; 4.18) in the full cohort compared to 6.92 (95% CI < 0; 16.59) in the 1960 + sub-cohort. The ERR/100 WLM decreased with increasing time since exposure, attained age and exposure rate in both cohorts. However, in the full cohort more than 25 years after exposure and more than 65 years of attained age no further decrease in risk was observed. This is also illustrated in Fig. 1. In the 1960 + sub-cohort, the confidence intervals of parameter estimates were very wide and did not indicate statistical significance. The estimated LEAR per WLM was about two times higher in the 1960 + sub-cohort compared to the full cohort with 6.10 × 10^–4^ and 3.13 × 10^–4^, respectively.

**Table 3:** Radon-related lung cancer risk according to BEIR VI exposure-age-concentration model (model 4) applied to the Wismut full cohort and 1960 + sub-cohort

	Full cohort	1960 + sub-cohort
Lung cancer deaths	4329	663
Person-years at risk	2,461,269	1,058,712
ERR/100 WLM (95% CI)	2.50 (0.81; 4.18)	6.92 (< 0; 16.59)
Time since exposure (years)		
5–14	1.0	1.0
15–24	0.96 (0.47; 1.46)	0.95 (< 0; 2.40)
25–34	0.64 (0.30; 0.97)	0.36 (< 0; 0.92)
35 +	0.61 (0.27; 0.94)	
Attained age (years)		
< 55	1.0	1.0
55–64	0.44 (0.27; 0.70)	0.83 (0.24; 2.84)
65–74	0.33 (0.20; 0.55)	0.34 (0.08; 1.52)
75 +	0.34 (0.19; 0.60)	0.09 (0.01; 5.89)
Exposure rate (WL)		
< 0.5	1.0	1.0
0.5–1.0	0.60 (0.36; 0.99)	0.90 (0.50; 1.64)
1.0–3.0	0.40 (0.26; 0.61)	0.57 (0.20; 1.62)
3.0–5.0	0.34 (0.22; 0.53)	–
5.0–15	0.28 (0.18; 0.44)	–
15 +	0.15 (0.08; 0.26)	–
LEAR per WLM (× 10^4^)	**3.13**	**6.10**

Baseline stratified by attained age, calendar year and duration of employment

Values of LEAR per WLM (× 10^4^) (bold)

*ERR* excess relative risk, *CI* confidence interval, *WLM* working level months, *WL* working level, LEAR lifetime excess absolute risk (exposure of 2 WLM from age 18 to 64 years, maximum age 94 and ICRP Euro-American-Asian mixed population)

Table 4 provides information on the estimated fraction of lung cancer deaths attributable to occupational radon among exposed miners by category of cumulative radon exposure, calendar year of death and attained age based on the BEIR VI model. In the full cohort, a total of 47% of all lung cancer deaths are estimated to be attributable to occupational radon exposure, i.e. 1853 out of 3956 lung cancer deaths could have been avoided without this exposure. In the 1960 + sub-cohort the attributable fraction is 31%. The attributable fraction increases with increasing cumulative radon exposure; for example, in the exposure category 1500 WLM or more 91% of the observed lung cancer deaths are estimated to be attributable to occupational radon. There is a clear decrease of attributable lung cancer deaths with increasing calendar year of death and increasing age in both, the full cohort and the 1960 + sub-cohort, reflecting the decrease in risk with increasing time since exposure and attained age. Importantly, 1 in 4 lung cancer deaths in the full cohort (26%) are still estimated to be attributable to occupational radon exposure and 1 in 5 lung cancer deaths of miners first hired in 1960 or later (19%) (see Appendix Fig. 2).

**Table 4:** Estimated excess lung cancer deaths due to radon exposure according to the BEIR VI exposure-age-concentration model (model 4) applied to the full cohort and 1960 + sub-cohort

	Full cohort			1960 + sub-cohort		
Lung cancer deaths	Excess *n*^b^	Observed *n*	Attributable fraction	Excess *n*^b^	Observed *n*	Attributable fraction
0 WLM	0	373	–	0	112	–
> 0 WLM^a^	1853	3956	46.8	171	551	31.0
Cum. radon exp. (WLM)						
> 0–10	12	442	2.7	15	179	8.4
10–50	63	442	14.3	75	230	32.6
50–100	59	242	24.4	51	98	52.0
100–500	387	912	42.4	30	44	68.2
500–1000	602	964	62.4	–	–	–
1000–1500	430	623	69.0	–	–	–
1500 +	300	331	90.6	–	–	–
Calendar year of death						
< 1960	8	15	53.3	–	–	–
1960–1970	145	197	73.6	–	–	–
1970–1980	378	577	65.5	5	8	62.5
1980–1990	453	816	55.5	17	29	58.6
1990–2000	432	949	45.5	36	71	50.7
2000–2010	290	838	34.6	65	191	34.0
2010–2018	146	564	25.9	48	252	19.0
Attained age (years)						
< 45	72	113	63.7	14	27	51.9
45–55	309	461	67.0	34	80	42.5
55–65	566	1166	48.5	79	218	24.8
65–75	566	1391	40.7	40	183	21.9
75 +	339	825	41.1	3	43	7.0

*WLM* working level months

^a^5 year lagged

^b^Small deviations in totals possible due to rounding

**Fig. 2:**
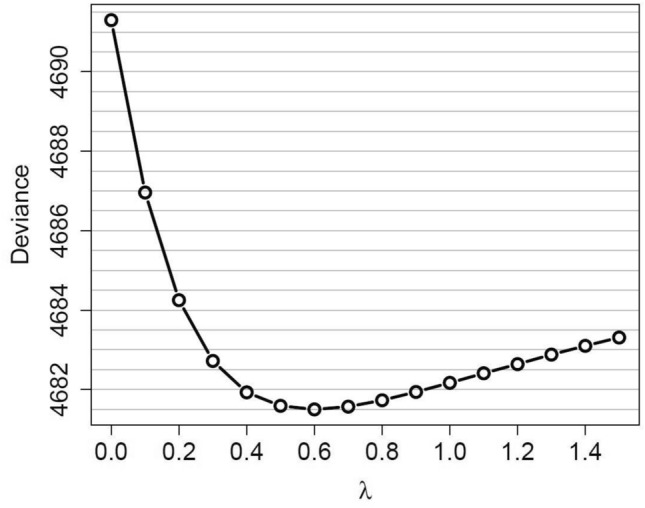
Deviance obtained when modelling the interaction of radon exposure and smoking related to lung cancer mortality depending on mixing parameter *λ*, based on geometric mixture models (GMM)

Some information on smoking is available for 56% of the 1960 + sub-cohort. Among those with known smoking status, 42% were non-/light smokers and 58% moderate/heavy smokers, the corresponding numbers among lung cancer deaths were 10% (*n* = 33), and 90% (*n* = 297), respectively. In a separate analysis among both groups, with the simple linear model, the ERR/100 WLM was 1.77 (95% CI < 0; 5.04) and 1.06 (95% CI 0.28; 1.85) among non-/light and moderate/heavy smokers, respectively. The slightly higher ERR/100 WLM for non/-light smokers compared to moderate/heavy smokers indicates a sub-multiplicative interaction of radon and smoking. The nature of this interaction was investigated in more detail by fitting GMM models for different values of the mixing parameter *λ* that determines the type of interaction as in Kreuzer et al. (2018). The minimum deviance was achieved for *λ* = 0.6, indicating a sub-multiplicative interaction (Fig. 2).

## Discussion

The updated findings of the Wismut cohort of uranium miners confirmed the previously observed linear relationship between the relative lung cancer risk and cumulative exposure to radon progeny, which was modified by time since exposure, attained age and exposure rate in the full cohort (Kreuzer et al. 2018). The ERR/WLM decreased with increasing time since exposure, attained age and exposure rate, both for parametric and BEIR VI-type models. For the first time, a statistically significant effect modification of the ERR/WLM by attained age and time since exposure was found in the 1960 + sub-cohort. Notably the ERR/WLM and corresponding LEAR per WLM were about two times higher in the 1960 + sub-cohort compared to the full cohort in models including effect modifiers. For example, when the BEIR VI exposure-age-concentration model was applied, the ERR/100 WLM among miners with attained age < 55 years, time since exposure 5–14 years, and annual exposure rates < 0.5 WL was 2.50 (95% CI 0.81; 4.18) compared to 6.92 (95% CI < 0; 16.59) in the full cohort and in the 1960 + sub-cohort, respectively. The same was true for the parametric model (model 3). Here, the ERR/100 WLM was 2.83 (95% CI 1.57; 4.09) compared to 5.38 (95% CI 1.76; 8.99), respectively, for exposure rates below 0.5 WL, 30 years of age at median exposure and 20 years of time since median exposure.

### Comparison with previous Wismut findings

Previous estimates of lung cancer risk in the Wismut cohort had been slightly lower compared to those of the current analyses for both the full cohort and the 1960 + sub-cohort (Kreuzer et al. 2015, 2018). One of the reasons for this is that previously no baseline stratification for duration of employment was applied. Consistent with findings in the PUMA analyses (Kelly-Reif et al. 2023; Richardson et al. 2022) this stratification was added in the current analyses and led also here to an increase in risk estimates. For example, for the BEIR VI model in the full cohort, the reference ERR/100 WLM with and without additional stratification was 2.50 versus 1.62 and the corresponding LEAR per WLM 3.13 × 10^–4^ versus 2.08 × 10^–4^, respectively (see Appendix Table 1). This effect was also present in the 1960 + sub-cohort, here the ERR/100 WLM in the simple linear model was 1.34 (95% CI 0.75; 1.93) versus 0.94 (95% CI 0.51; 1.37) with and without stratification for duration of employment (Appendix Table 2), and the LEAR per WLM in the finally preferred parametric model 2 (including two modifiers) 7.13 × 10^–4^ and 4.12 × 10^–4^, respectively. An explanation for the strong effect of stratification by duration of employment could be the healthy worker survivor effect (Keil et al. 2015), which means that healthy workers are more likely to work for a long time underground and thus accumulate high radon exposures compared to those who have health problems and change their workplace, e.g. from underground to surface or elsewhere. This could artificially lead to lower risks among long-term workers compared to short-term workers. The Wismut cohort is characterized by a wide range of employment durations, around 25% of the cohort members worked for more than 20 years and 13% for more than 30 years in the company.

Due to the young age of the 1960 + sub-cohort, no statistically significant effect modification of the ERR/WLM by attained age or time since exposure has been found previously (Kreuzer et al. 2015, 2018). Such an effect modification was demonstrated in the large PUMA 1960 + sub- cohort (Richardson et al. 2022), and now also in the current extended follow-up until 2018 in the Wismut 1960 + sub-cohort. However, even in the current follow-up there are only 43 lung cancer deaths among radon exposed miners with an age above 75 years (see Table 4), therefore risk estimates for longer times since exposure and higher attained ages involve uncertainties. There is some evidence that exposure rate might be an additional modifier in the 1960 + sub-cohort, although the model including this factor was not statistically significantly better than the model without this factor (Table 2, model 3 vs. model 2).

### Differences in risk between full Wismut cohort and 1960 + sub-cohort

There are two to three times higher risk estimates at low exposures and exposure rates in the 1960 + sub-cohort compared to the full cohort, which require further clarification. Several reasons may account for this. Firstly, uncertainties in the assessment of radon exposure in the early years may have led to an underestimation of the true risk in the full cohort. The job-exposure matrix was based on expert rating rather than measurements in the early years of mining in the Wismut company, thus bias due to exposure measurement error is of concern. In some uranium miners cohort studies risk estimates were higher when focussing on miners in more recent years compared to earlier years (Tomasek et al. 2008; UNSCEAR 2020; Lane et al. 2019). This fact has sometimes been attributed to lower quality of exposure assessment and a potentially higher impact of measurement error on the risk estimation in the early years. Currently, a research project (Küchenhoff et al. 2018; Ellenbach et al. 2023) is running to investigate sources, magnitude and potential effects of exposure measurement error in the Wismut cohort. Similar investigations have been performed in the Colorado Plateau (Stram et al. 1999), Ontario (Navaranjan et al. 2019) or French (Hoffmann et al. 2017) cohort of uranium miners.

Secondly, the mortality follow-up was rather incomplete in the early years. For example, the proportion of loss to follow-up was 10% versus 2% for those with end of employment before 1960 or later, respectively; this was mainly because the persons could no more be identified by the registration offices under the last known address from the 1950s. The proportion of missing causes of death before 1970 was 44% compared with 3.5% later; the main reason was that copies of death certificates from before 1970 were often no more available. For the early years in the full cohort, the incomplete mortality follow-up concerned particularly miners with young age at death and short time of follow-up—the factors associated with the highest risk. Thirdly, in the early mining years, exposures to both silica dust and radon were extremely high, therefore the risk of lung cancer may be underestimated in the full cohort due to the competing risk of dying from silicosis (e.g. a total of 1,067 miners died from silicosis as underlying cause of death in the full cohort, in contrast to only 11 silicosis deaths in the 1960 + sub-cohort).

Another reason for differences in risk estimates between the full and 1960 + sub-cohort could be overestimation of risk in the 1960 + sub-cohort. Time since exposure turned out to be a strong modifier in most studies of miners, showing that the ERR/WLM is highest 5–15 years after exposure and decreases with increasing time since exposure (NRC 1999; UNSCEAR 2009, 2020). In the 1960 + sub-cohort, the duration of follow-up is shorter than for miners hired prior to 1960. In addition, average age is appreciably younger (Table 1). Consequently, the decrease in risk with increasing time since exposure and attained age cannot be completely described by the data of the 1960 + sub-cohort. This is illustrated in the 1960 + sub-cohort when risk estimates from the BEIR VI model are compared for end of follow-up by 2013 and 2018. In analyses with end of follow-up in 2013 an increase in risk is observed in age category 75 + years (Appendix Table 3), which is also seen in risk predictions in Appendix Fig. 4. These findings indicate that the higher LEAR per WLM of 9.22 × 10^–4^ in analyses based on data with end of follow-up in 2013 compared to 6.10 × 10^–4^ with end of follow-up in 2018 (Appendix Table 3), resulted from a lack of decrease in risk after 75 years of age.

### Comparison with PUMA findings

The PUMA study includes seven uranium cohorts of miners from Europe and North America, among them the Wismut cohort with mortality follow-up by end of 2013 excluding millers (Rage et al. 2020; Richardson et al. 2021). Two papers on the lung cancer risk by radon have been published by now, one on the full cohort (Kelly-Reif et al. 2023) and one on the 1960 + sub-cohort (Richardson et al. 2022). In the full PUMA cohort, a reference ERR/100 WLM of 4.68 (95% CI 2.88; 6.96) was observed for the BEIR VI exposure-age-concentration model (Kelly-Reif et al. 2023). However, a statistically significant heterogeneity between cohorts was present, which was in part attributable to the comparably lower risk in the full Wismut cohort, which forms about half of the PUMA cohort. The PUMA 1960 + sub-cohort did not show such heterogeneity between study cohorts, here the corresponding reference ERR/100 WLM was 6.98 (95% CI 1.97; 16.15) (Richardson et al. 2022), which is consistent to the estimates of the updated Wismut 1960 + sub-cohort of 6.92 (95% CI< 0, 16.59), of the pooled 11 miners study of 7.68 (NRC 1999) and others (Lane et al. 2010, 2019). Lifetime risk calculations for lung cancer and radon within PUMA are planned by the PUMA consortium in a separate paper.

### Interaction of radon and smoking

The present analysis indicated a sub-multiplicative interaction of radon and smoking in the 1960 + sub-cohort via GMM modelling. This finding is supported by the results of a simple linear model separately for both smoking groups, here the ERR/100 WLM in non-/light smokers was slightly higher compared to moderate/heavy smokers (1.77 versus 1.06). Previous analyses based on the last Wismut follow-up (Kreuzer et al. 2018) also found higher ERR/100 WLM among non-/light smokers compared to moderate/heavy smokers (2.0 versus 1.2), however at that time GMM modelling indicated rather a multiplicative to supra-multiplicative interaction. Statistical uncertainty due to a small number of lung cancer deaths among non-/light smokers (*n* = 33) is still of concern. Further follow-up may bring more insights into the interaction of radon and smoking. A sub-multiplicative interaction is compatible with the findings from most other miner studies, while in residential radon studies, no obvious deviation from a multiplicative interaction has been consistently observed (UNSCEAR 2020).

### Strengths and weaknesses of the study

The Wismut cohort is unique due to its large size, long follow-up period from 1946 to 2018, wide range of exposures and availability of individual data not only on radon exposure, but also on silica dust, long-lived radionuclides and external gamma radiation as well as in the 1960 + sub-cohort in part on rough data on smoking. Detailed investigation of potential confounding of the radon-related lung cancer risk by these factors have been performed previously in the full cohort (Walsh et al. 2010) and in a nested case–control study on lung cancer with smoking data (Schnelzer et al. 2010). Overall, none of the above-mentioned variables led to major confounding, except for silica dust, here a 25% decrease in lung cancer risk estimates was observed after including silica dust in the risk model (Walsh et al. 2010). In the 1960 + sub-cohort, confounding could be even more relevant due to the lower occupational radon exposure and thus smaller radon-related lung cancer risk. As shown in Appendix Table 2 additional adjustment for smoking and external gamma radiation in model 2 resulted in slight decreases in the risk estimates and thus no major confounding. The mean cumulative occupational silica dust exposure was 1.0 mg/m^3^-years in the 1960 + sub-cohort in contrast to 6 mg/m^3^-years in the full cohort or even 12 mg/m^3^-years among miners first hired between 1946 and 1954, and is thus far below the threshold of 10 mg/m^3^, above which a silica dust related lung cancer risk was observed in the full Wismut cohort (Sogl et al. 2012).

The Wismut study includes next to underground and surface workers also millers and open pit miners, which differ by working conditions and are characterised by very low radon exposures. Exclusion of millers and open pit miners did not lead to a major change in risk, as shown in the sensitivity analysis presented in Appendix Table 1. Short-term workers (< 5 years of duration of employment) may differ in risk from long-term workers, as reflected by the healthy worker survivor effect. In addition to baseline stratification by duration of employment, excluding this group in sensitivity analyses showed virtually no change in risk (Appendix Tables 1 and 2). Exposure measurement error in the early years is an issue, as noted above, and the potential influence on risk is currently investigated.

## Conclusion

The updated Wismut cohort study shows an increased lung cancer risk by radon for former miners even 20 to 30 years after the mines were closed and occupational radon exposures ended. The estimated lifetime excess absolute risk (LEAR per WLM) to die from lung cancer per unit of cumulative radon exposure in WLM varies between 3 and 7 × 10^–4^ depending on model and (sub-) cohort, i.e. among 100 people with a cumulative occupational radon exposure of 100 WLM between 3 and 7 additional (excess) lung cancer deaths would occur due to this exposure during lifetime. The 1960 + sub-cohort is characterized by low protracted radon exposure of high quality and provides now, through the extension of follow-up to end of 2018, a good basis for the estimation of lung cancer risks at low radon exposures and low exposure rates. Risk estimates from the 1960 + sub-cohort are consistent with those from 1960 + sub-cohorts of large pooled studies. The Wismut 1960 + sub-cohort is thus preferred to the full Wismut cohort in order to estimate lung cancer risks at low protracted exposure rates for more contemporary miners, although the statistical power is lower than in the full cohort. Further follow-up of the Wismut 1960 + sub-cohort and pooled analyses of updated individual cohorts of PUMA will increase precision, particularly for attained ages above 75 years and longer times since exposure, this will lead to a better understanding of the lung cancer risk at low radon exposures and exposure rates.

### Supplementary Information

Below is the link to the electronic supplementary material.

Supplementary file1 (DOCX 116 KB)

## Data Availability

Access to the data can be obtained after a positive evaluation of the proposal by the Steering Committee on the German Uranium Mining Studies of the German Radiation Protection Commission (SSK) and the German Federal Office for Radiation Protection (BfS). The procedure of opening of the Wismut data to external researchers is described here: https://www.bfs.de/EN/bfs/science-research/projects/wismut/wismut-cohort-proposals.html.
