# Supplementary material for: Updated risk models for lung cancer due to radon exposure in the German Wismut cohort of uranium miners, 1946–2018
Source: Radiat Environ Biophys. 2023 Sep 11;62(4):415–25. doi: 10.1007/s00411-023-01043-2 (PMC10628028; doi:10.1007/s00411-023-01043-2)
Supplement: Supplementary file 1 — Supplementary file1 (DOCX 116 KB) [file 411_2023_1043_MOESM1_ESM.docx]

**Appendix Table 1:** Radon-related lung cancer risk according to the BEIR VI exposure-age-concentration model (model 4) applied to the full Wismut cohort – sensitivity analyses

|  | **Model 4,  full cohort** | **Model 4*, full cohort without baseline stratification for duration of employment** | **Model 4, full cohort  without persons with duration of employment <5 years** | **Model 4, full cohort  without millers and open pit miners** |
| --- | --- | --- | --- | --- |
| Lung cancer deaths | 4,329 | 4,329 | 3,556 | 4,022 |
| Person-years at risk | 2,461,269 | 2,461,269 | 1,767,825 | 2,210,227 |
|  |  |  |  |  |
| ERR/100 WLM (95% CI) | 2.50 (0.81; 4.18) | 1.62 (0.54; 2.70) | 2.23 (0.66; 3.81) | 2.19 (0.59; 3.79) |
|  |  |  |  |  |
| Time since exposure (years) |  |  |  |  |
| 5-14 | 1.0 | 1.0 | 1.0 | 1.0 |
| 15-24 | 0.96 (0.47; 1.46) | 0.91 (0.45; 1.38) | 1.14 (0.51; 1.77) | 0.92 (0.42; 1.42) |
| 25-34 | 0.64 (0.30; 0.97) | 0.66 (0.33; 1.00) | 0.78 (0.34; 1.22) | 0.58 (0.25; 0.91) |
| 35+ | 0.61 (0.27; 0.94) | 0.62 (0.29; 0.95) | 0.68 (0.28; 1.08) | 0.52 (0.21; 0.84) |
| Attained age (years) |  |  |  |  |
| <55 | 1.0 | 1.0 | 1.0 | 1.0 |
| 55-64 | 0.44 (0.27; 0.70) | 0.48 (0.31; 0.72) | 0.43 (0.25; 0.71) | 0.47 (0.27; 0.79) |
| 65-74 | 0.33 (0.20; 0.55) | 0.34 (0.22; 0.53) | 0.34 (0.20; 0.58) | 0.43 (0.25; 0.75) |
| 75+ | 0.34 (0.19; 0.60) | 0.34 (0.20; 0.56) | 0.35 (0.19; 0.65) | 0.40 (0.21; 0.76) |
| Average exposure rate (WL) |  |  |  |  |
| <0.5 | 1.0 | 1.0 | 1.0 | 1.0 |
| 0.5-1.0 | 0.60 (0.36; 0.99) | 0.71 (0.39; 1.28) | 0.60 (0.36; 0.98) | 0.60 (0.35; 1.02) |
| 1.0-3.0 | 0.40 (0.26; 0.61) | 0.51 (0.31; 0.84) | 0.40 (0.26; 0.60) | 0.41 (0.26; 0.66) |
| 3.0-5.0 | 0.34 (0.22; 0.53) | 0.45 (0.27; 0.74) | 0.34 (0.22; 0.53) | 0.36 (0.22; 0.57) |
| 5.0-15 | 0.28 (0.18; 0.44) | 0.38 (0.23; 0.63) | 0.29 (0.19; 0.44) | 0.30 (0.19; 0.29) |
| 15+ | 0.15 (0.08; 0.26) | 0.21 (0.11; 0.39) | 0.15 (0.08; 0.27) | 0.16 (0.08; 0.29) |
| **LEAR per WLM (×10^4^)** | **3.13** | **2.08** | **3.22** | **2.97** |

ERR: Excess Relative Risk, CI: Confidence Interval, Baseline stratified by attained age, calendar year and duration of employment
WLM: Working Level Months, WL: Working Level
LEAR: Lifetime Excess Absolute Risk (exposure of 2 WLM from age 18 to 64 years, maximum age 94 and ICRP Euro-American-Asian mixed population).
* Baseline stratified by attained age and calendar year only

**Appendix Table 2:** Radon-related lung cancer risk according to parametric models applied to the Wismut 1960+ sub-cohort (Models 1 and 2) – sensitivity analyses

|  |  | **1960+ sub-cohort** | | | | |
| --- | --- | --- | --- | --- | --- | --- |
| **Models** | **Para-meter** | **Models 1+2** | **Models 1+2*  without baseline stratification for duration of employment*** | **Models 1+2*,  without persons with duration of employment <5 years** | **Models 1+2 adjusted for external γ-radiation** | **Models 1+2, adjusted for smoking in three categories** |
| Lung cancer deaths |  | 663 | 663 | 476 | 663 | 663 |
| Person-years at risk |  | 1,058,712 | 1,058,712 | 702,512 | 1,058,712 | 1,058,712 |
| Model 1 |  |  |  |  |  |  |
| ERR/100 WLM (95% CI) | *ß* | 1.34 (0.75; 1.93) | 0.94 (0.51; 1.37) | 1.35 (0.74; 1.96) | 1.05 (0.42; 1.68) | 1.04 (0.52; 1.56) |
| **LEAR per WLM (×10^4^)** |  | **6.09** | **4.27** | **6.13** | **4.77** | **4.73** |
|  |  |  |  |  |  |  |
| Model 2 |  |  |  |  |  |  |
| ERR/100 WLM (95% CI) | *ß* | 4.66 (1.71; 7.62) | 2.08 (0.75; 3.41) | 3.35 (1.03; 5.67) | 4.15 (1.29; 7.02) | 3.34 (1.06; 5.61) |
| Age at median exposure | exp(10*α*) | 0.74 (0.44; 1.26) | 0.79 (0.42; 1.49) | 0.90 (0.52; 1.56) | 0.71 (0.39; 1.31) | 0.76 (0.42; 1.36) |
| Time since median exposure | exp(10*ε*) | 0.47 (0.30; 0.73) | 0.59 (0.38; 0.93) | 0.57 (0.36; 0.90) | 0.45 (0.27; 0.75) | 0.50 (0.31; 0.80) |
| *p*-value (Model 2 vs. 1) |  | <0.001 | 0.044 | 0.011 | 0.002 | 0.006 |
| **LEAR per WLM (×10^4^)** |  | **7.13** | **4.12** | **7.27** | **5.91** | **5.50** |

ERR: Excess Relative Risk, CI: Confidence Interval, Baseline stratified by attained age, calendar year and duration of employment
WLM: Working Level Months, WL: Working Level
*p*-value of likelihood ratio test between the two nested models
LEAR: Lifetime Excess Absolute Risk (exposure of 2 WLM from age 18 to 64 years, maximum age 94 and ICRP Euro-American-Asian mixed population).
* Baseline stratified by attained age and calendar year only

**Appendix Table 3:** Radon-related lung cancer risk according to the BEIR VI exposure-age-concentration model for the Wismut 1960+ sub-cohort with end of follow-up 2013 vs. 2018

| **End of follow-up** | **31/12/2013** | **31/12/2018** |
| --- | --- | --- |
| Lung cancer deaths | 495 | 663 |
| Person-years at risk | 956,776 | 1,058,712 |
|  |  |  |
| ERR/ 100 WLM (95% CI) | 7.13 (<0; 16.84) | 6.92 (<0; 16.59) |
|  |  |  |
| Time since exposure (years) |  |  |
| 5-14 | 1.0 | 1.0 |
| 15-24 | 0.79 (<0; 2.03) | 0.95 (<0; 2.40) |
| 25-34 | 0.35 (<0; 0.88) | 0.36 (<0; 0.92) |
| 35+ |  |  |
| Attained age (years) |  |  |
| <55 | 1.0 | 1.0 |
| 55-64 | 0.90 (0.25; 3.18) | 0.83 (0.24; 2.84) |
| 65-74 | 0.42 (0.09; 1.97) | 0.34 (0.08; 1.52) |
| 75+ | 0.49 (0.04; 5.62) | 0.09 (0.01; 5.89) |
| Exposure rate (WL) |  |  |
| <0.5 | 1.0 | 1.0 |
| 0.5-1.0 | 0.85 (0.46; 1.57) | 0.90 (0.50; 1.64) |
| 1.0+ | 0.31 (0.06; 1.53) | 0.57 (0.20; 1.62) |
| **LEAR per WLM (**×**10^4^)** | **9.22** | **6.10** |

ERR: Excess Relative Risk, CI: Confidence Interval
WLM: Working Level Months, WL: Working Level
LEAR: Lifetime Excess Absolute Risk (exposure of 2 WLM from age 18 to 64 years, maximum age 94 and ICRP Euro-American-Asian mixed population).
Baseline stratified by attained age, calendar year and duration of employment

**Appendix Figure 1**: Mean annual exposure to radon progeny in WLM among exposed persons in the full Wismut cohort (dashed line) and the 1960+ sub-cohort of miners first hired in 1960 or later (solid line with diamonds).

**Appendix Figure 2**: Estimated background and excess lung cancer deaths due to radon exposure per category of calendar year according to the BEIR VI exposure-age-concentration model (model 4) in the full cohort and the 1960+ sub-cohort (see also Table 4)


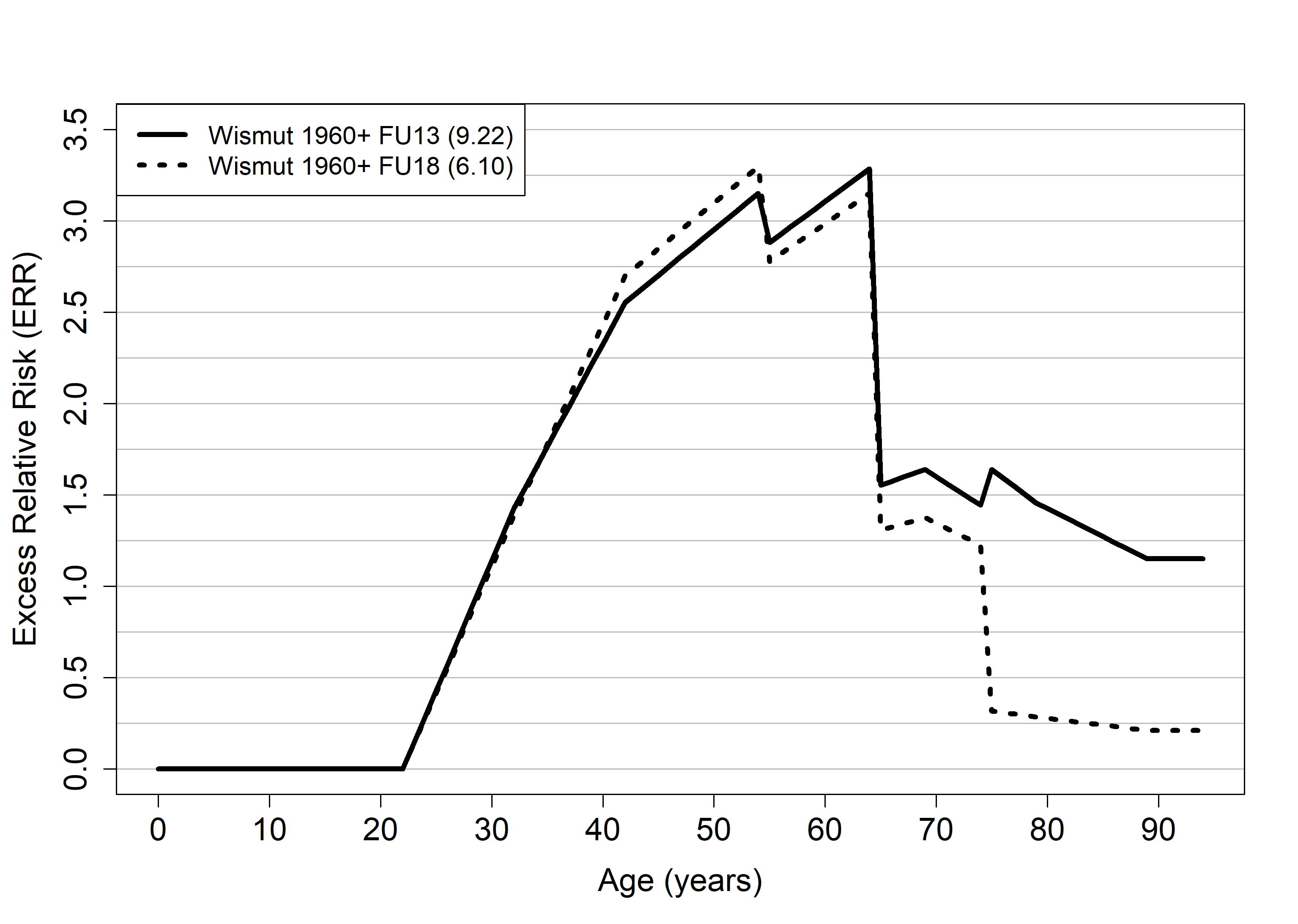


**Appendix Figure 3**: Excess relative risk predicted in the Wismut 1960+ sub-cohort with end of follow-up 2013 (solid line) and end of follow-up 2018 (dashed line) for the exposure scenario of 2 WLM from age 18 to 64 up to age 94 assuming a 5-year lag based on the BEIR VI exposure-age-concentration model with corresponding total LEAR in brackets in figure legend, respectively.
